# Gastroprotective Effects of Astragaloside IV against Acute Gastric Lesion in Rats

**DOI:** 10.1371/journal.pone.0148146

**Published:** 2016-02-04

**Authors:** Shuai Mao, Guang Yang, Winny Li, Jian Zhang, Hailong Liang, Jian Li, Minzhou Zhang

**Affiliations:** 1 Department of Critical Care Medicine, Guangdong Provincial Hospital of Chinese Medicine, Road Dade, Guangzhou 510120, China; 2 Second Clinical Medical College, Guangzhou University of Chinese Medicine, Road Jichang, Guangzhou 510405, China; 3 Faculty of Medicine, University of Toronto, University Ave., Toronto M5G 0A4, Canada; 4 Physiology & Experimental Medicine, Hospital for Sick Children, Toronto M5G 0A4, Canada; Panjab University, INDIA

## Abstract

**Background:**

Protection of the gastric mucosa from acute lesions induced by various irritants is a pertinent issue in the field of critical care medicine. In this study, we investigated the gastroprotective effects of *astragaloside IV* on acute gastric lesions in rats under stressful conditions.

**Methods:**

Rats were randomized into six groups. Group 1 and 2 received 10% Tween 80 (vehicle). Group 3 received 20 mg/kg of omeprazole, a proton pump inhibitor. Groups 4, 5 and 6 received *astragaloside IV* at concentration of 1, 10, and 50 mg/kg, respectively. As a means to induce gastric lesions, Groups 2–6 were subjected to water immersion and restraint stress for 12 hours after treatment.

**Results:**

Our present studies show that compared to rats in group 2, treatment with 1 to 50 mg/kg *astragaloside IV* significantly decreased the size of gastric lesions, MDA, TNFα and MCP1 levels, in addition to normalizing gastric pH, gastric mucus and SOD levels (P<0.05). Histomorphological examination confirmed that treatment with *astragaloside IV* elicited a dosage-dependent protective effect on the gastric mucosa. Furthermore, pretreatment with *astragaloside IV* resulted in significant elevations in HSP70 and reduction in Bax, along with over-expression of PLCγ response level, which was further confirmed via immunohistochemical analysis.

**Conclusions:**

The acute gastric lesions induced are attenuated by pretreatment with *astragaloside IV* which is possibly due to the enhancing of the expression of HSP70 with concomitant antioxidant, anti-inflammatory and anti-apoptotic capacity.

## Introduction

Stress-induced gastric damage is a common occurring disease affecting a large number of patients of all ages, races and socio-economic classes throughout the world [1]. Clinically, stress induced gastritis can cause mucosal damage, resulting in hemorrhagic erosions. The overall incidence of developing stress-induced acute gastric lesion secondary to other serious clinical conditions is as high as 1.5% and is usually associated with significantly higher morbidity and mortality [2]. The pathophysiological mechanism responsible for acute gastric lesion development involves inflammatory infiltration, endothelial dysfunction, free radical oxygen mediated cell membrane damage in response to mucosal damage [3]. Despite the number of treatment strategies, including reduction of gastric acid secretion responsible for erosion of the gastrointestinal mucosa, the long-term prognosis of acute gastric lesion remains poor [4]. Moreover, many of the pharmacologic agents currently use to treat acute gastric lesions is associated with adverse side effects such as: arrhythmias, nausea, dizziness, upper respiratory infection, hypergastrinemia, constipation, and metabolic alkalosis [5]. Together, this highlights the need for the discovery and validation of alternative natural agents that confer gastric mucosa protection and consequently reduce the incidence of the acute gastric lesions in critical diseases, without inducing the remote complications.

Over the past few years, natural products from plants and herbs have received a great deal of attention, largely due to the discovery of their potential effectiveness in alleviating symptoms of gastric ulcers, gastrointestinal bleeding, bowel obstruction, acute abdomen and other gastrointestinal conditions [6–10]. *Astragaloside IV* in particular, is a well-known traditional Chinese herbal medicine that is extracted from the dried root of *leguminous plants Mongolia Astragalus* [*Astragalus membranaceus (Fisch*.*) Bge*.]. In countries like China, Korea, Malaysia and Japan, modern clinical practice continues to rely on *astragaloside IV*-derived preparations for its induction of anti-inflammatory, antioxidant and immunoenhanced effects [11, 12]. Experimental animal studies have shown *astragaloside IV* to have vasodilatation effects following nitric oxide activation and protected against endothelial dysfunction, which contributed to the reduction of mucosal ischemia in rodents exposed to experimental sepsis induced by cecal ligation and puncture [13]. *Astragaloside IV* also exhibits anti-inflammatory effects by lowering endogenous levels of prostaglandins while inhibiting secretion of proinflammatory cytokines such as interleukin1β and tumor necrosis factor α (TNFα) [14]. Treatment with *astragaloside IV* also alleviated the development of gastrointestinal failure and decreased the mortality in experimental septic rabbits [15]. Recent reports showed that the *astragaloside IV* exerts an antispasmodic effect on the rat ileum and provokes anti-nociceptive actions [16]. Our previous study demonstrated that *astragalus membranaceus* exhibited cytoprotecting action against indomethacin-induced gastric lesions in rats.

While the ultimate beneficial modulations in gastrointestinal function have been reported in the *astragaloside IV*-treated animals, the mechanisms of acute gastric lesion prevention and healing by this phytomedicine has not yet been explained. Thus, the present work aims to investigate the mechanisms of the gastroprotective effects of *astragaloside IV*. In this study water immersion and restraint stress (WRS) was performed to established an acute gastric lesion model and the gastro-protective action of *astragaloside IV* were assessed.

## Materials and Methods

### Ethics statement

Male Sprague-Dawley (SD) rats, weighing 180-220g, aged 6 weeks were purchased from Guangdong Medical Laboratory Animal Center. All experiments were carried out in accordance with an Institutional Ethics Committee approved protocol at Guangdong Provincial Hospital of Chinese Medicine (Ethic No. GZH/02/09/2011). We confirmed that the Institutional Animal Care and Use Committee (IACUC) or ethics committee specifically approved this study. Guidelines for the Declaration of Helsinki Principles and United States Institute of Animal Research guidelines for the care and use of laboratory animals were abided by throughout the duration of the study. Animals received humane care and were sacrificed under anesthesia with xylazine/ketamine (0.5 mL of xylazine [2 mg/mL] and 1 mL of ketamine [50 mg/mL]) at a dosage of 1.5 mL/100 g body weight.

### Reagents

Rat superoxide dismutase (SOD), malondialdehyde (MDA), monocyte chemotactic protein 1 (MCP1), tumor necrosis factors-α (TNF-α), heat-shock protein (HSP70), BCL2-associated X protein (BAX), phospholipase Cγ (PLCγ) and extracellular signal-regulated kinase 1/2 (ERK1/2) Enzyme-linked immunosorbent assay kit were purchased from R&D Systems Inc. (Minneapolis, MN, USA). *Astragaloside IV* (purity 99.1%) and Omeprazole (purity 99.9%) were purchased from Chinese Institute for Drug and Biological Product Control (Beijing, China). RNeasy Mini Kit for isolating total RNA was purchased from GE Healthcare (Buckinghamshire, UK). The cDNA was synthesized using SuperScript™ II RT obtained from Qiagen Inc. (Mississauga, ON, Canada). Relative quantification of mRNA gene expression was performed using SYBR^®^ Green PCR Assays (Applied Biosystems, Foster City, CA). All other chemical grade reagents were purchased from Skreda Science & Technology Development Co.Ltd (Beijing, China).

### Grouping and treatment

Forty-eight SD rats were used in this study. The animals were fasted for 24 hours with free access to water that was removed three hours prior to the start of pretreatment. Experimental rats were kept at 24°C in humidity-controlled rooms on a standard light/dark cycle.

The animals were divided equally into six groups (eight rats each) according to the following experimental design.

Group 1: normal blank control (negative control), received 5mL/kg bow. of 10%Tween 80 (vehicle) by gavage.

Group 2: gastric lesions control, received 5 mL/kg bow. of 10% Tween 80 by gavage.

Group3: positive control, received 5 mL/kg bow. of omeprazole (20 mg/kg, suspended in 10%Tween 80), a known proton pump inhibitor, by gavage.

Group 4, 5 and 6 were intragastrical administered with5 mL/kg bow. of *astragaloside IV* at a dosage of 1, 10, and 50 mg/kg (suspended in 10%Tween 80), respectively.

One hour after pretreatment, acute gastric lesions were introduced to Groups 2 to 6 by exposing animals to WRS at 20°C according to the procedure described by Brzozowski [17]. The animals were also kept in Bollman-type cages to induce restrain stress. The experimental conditions in this study were modeled to mimic the elevated stress response that occurs in the clinical setting of trauma, sepsis or shock.12 hour after modeling, all animals were sacrificed and the stomachs were immediately recovered for further analyses.

### Measurement of gastric acid and mucus production

The resected stomachs were immediately excised along the greater curvature, and the gastric acid pH was measured by metric titration using a Digital pH meter. Gastric pH measurements were made in three areas of the oxyntic mucosa not involving macroscopically visible lesions. The mean value of the measurements were calculated and expressed as a pH value.

Gastric mucus samples were gently scraped and collected from the gastric mucosa of each animal using a glass slide, and the samples were subsequently weighed using a precision electronic balance. The stomach samples were then washed with ice-cold PBS, and prepared for lesion area measurement and histopathological examinations.

### Evaluation of acute gastric lesions

Stomachs of experimental rats were excised along the greater curvature to assess the extent of gastric lesions in a double-blind fashion. The stress lesion was defined as a round or linear mucosal defect of at least 1 mm in diameter with hemorrhagic lesions. The length of each lesion along its greatest diameter and width were measured. The sum of all impaired areas for each stomach was expressed as the lesion area (mm^2^), which was calculated as described by Kauffman Jr. Grossman [18]. The inhibition percentage (%) was calculated according to the formula:

$$I\%\;=\;[(UA_{control}\;-\;UA_{treated})\;÷\;UA_{control}]\;\times \;100\;\%$$

### Histopathological evaluation of gastric lesions

Samples of the gastric walls from experimental rats were fixed in 10% formalin and subsequently paraffin embedded and 5 μm sections were prepared. The sections were stained with haematoxylin and eosin (H&E) and examined microscopically.

### Determination of protein response level

The gastric tissues were collected and washed with cold saline and subsequently homogenised in 5:1 (w/v) PBS (pH 7.4). After centrifugation at 25,000 x *g* for 15 min at 4°C, the supernatant was stored at -80°C prior to analysis. The homogenates were used for the estimation of superoxide dismutase (SOD), total antioxidant capacity (MDA), MCP1, TNF-α, HSP70, Bax, PLCγ and ERK1/2protein expression by enzyme-linked immunosorbent assay (ELISA) (R&D Sys., USA) according to the manufacturer’s protocol. In brief, 10*μ*L of samples and 200 *μ*L of diluted buffer were added to 96 wells plate following by shake for 1 min. 20 *μ*L of hydrogen oxidase dilution was added to each sample well and then incubated for 20 min at room temperature. 30*μ*L of potassium hydroxide dilution and 30 *μ*L of catalase purpald were successively added to the mixture and incubated for another 10 min on a shaker at room temperature. 10 *μ*L of catalase potassium periodate was added to each well and the sample was read using an ELISA reader.

### Measurement of HSP70 gene expression

Total RNA ofgastric tissue homogenates was isolated using the RNAspin Mini Kit (GE Healthcare, UK) according to the manufacturer’s instructions [19]. cDNA was synthesized using SuperScript™ II RT (Invitrogen, USA). PCR reaction carried out in a total reaction volume of 15 μL including: 7.5 μL of SYBR^®^ Green PCR master mix (Applied Biosystems, CA), 2 μL of cDNA template, and 0.2 μM of gene-specific primers.HSP70 primers used were: sense, 5’-AAGAGTTCCCCAGGGACCTC-3’ and antisense, 5’-GCTTGAGGGTTTGCTACAAC-3’. All samples were in duplicate and relative mRNA expression were determined using the comparative Ct method (ΔΔC_T_). Individual values were normalized by comparison to 18s mRNA.

### Immunohistochemical staining

Tissue sections were heated for 30 min in an oven at 60°C. The tissue sections were deparaffinized in xylene and rehydrated through an ethanol gradient. Antigen retrieval was performed using a sodium citrate buffer (10 mM) in a microwavable pressure cooker. Endogenous peroxidase was blocked using 0.03% hydrogen peroxide containing sodium azide. The tissue sections were washed and incubated with 2 μg/mL of polyclonal antibody to HSP70 (Santa Cruz, CA) for 1 hour. The sections were then incubated for an additional hour with appropriate fluorescein conjugated secondary antibody. Following antibody conjugation and washes, tissue sections were incubated with DAB-substrate chromagen for 5 min. The sections were then washed and counterstained with hematoxylin for 5 sec, then dipped in weak ammonia (0.037mol/L) 10 times and rinsed with distilled water prior to the mounting. Positive immunohistochemical staining was observed as a brown coloration in the tissue sections under a light microscope.

### Statistical analysis

All statistical analyses were performed with SPSS version 13.0 for Windows (SPSS, Inc. 2005). Macroscopic and microscopic data were compared by the Kruskal-Wallis nonparametric test, and other parameters were compared using a one-way ANOVA followed by Fisher’s least significant difference test. All results were expressed as means ± SD. Values of P < 0.05 or P < 0.01 were considered to be statistically significant.

## Results

### Astragaloside IV decreases gastric lesion areas in a dose-dependent manner

Results of the initial series of our experiments revealed that *astragaloside IV* attenuated acute gastric lesions induced by WRS in a dose-dependent manner. The animals in lesion control group (group 2) showed extensive and visible hemorrhagic injury in the gastric mucosa with lesion area 860.82 ±12.47 mm^2^ (Table 1).

**Table 1:** Effect of astragaloside IV on different gastric parameters of WRS-induced rats.

Groups	Treatment (5 mL/kg dose)	Lesion area (mm^2^)	Lesion inhibition (%)	pH value	Mucus production (mg)
1	Negative control	0	0	2.84±0.08*^#^	421.85±9.69*^#^
2	Lesion control	860.82±12.47^#^	0	2.05±0.09^#^	801.06±10.46^#^
3	Omeprazole (20 mg/kg)	270.58±9.13*	68.57	5.18±0.09*	1541.31±71.57*
4	astragaloside IV (1 mg/kg)	705.64±12.34*^#^	18.03	3.28±0.08*^#^	775.08±6.68^#^
5	astragaloside IV (10 mg/kg)	499.41±5.48*^#^	41.26	3.59±0.10*^#^	1106.62±15.13*^#^
6	astragaloside IV (50 mg/kg)	251.48±9.26*	70.79	4.63±0.16*^#^	1521.11±84.33*

All values were expressed as mean ± standard error of mean.

*Significant difference (*P*< 0.05) when compared with the lesion control (group 2).

^#^Significant difference (*P*< 0.05) when compared with the omeprazole (group 3).

Pretreatment of rats with *astragaloside IV* (groups 4–6) or omeprazole (the reference drug group) before exposure to WRS significantly reduced areas of gastric lesion when compared to group 2(P<0.05).

**Fig 1:**
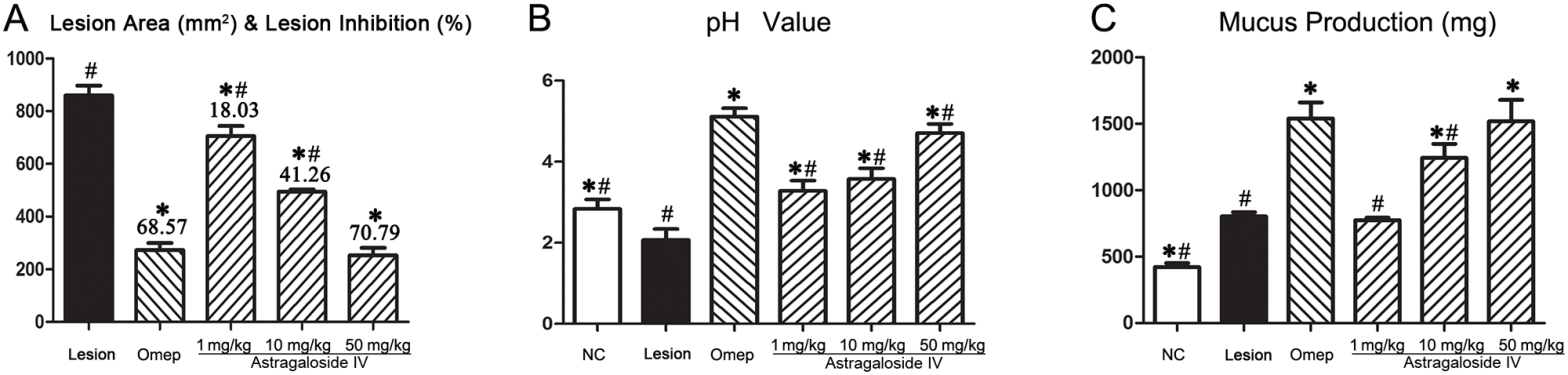
The effects of *astragaloside IV* treatment on the gastric lesions area, pH value and mucus production. Astragaloside IV or omeprazole were administered 1 h prior to WRS-induced gastric lesion to assess the protective effect. Lesion control group rats received Tween 80. After 12h, rats were sacrificed and lesion area, pH value and mucus production were measured. NC, negative control; Lesion, Lesion control; Ome: omeprazole. Data expressed as mean ± standard error of mean. *Significant difference (*P*< 0.05) when compared with the lesion control (group 2), ^#^Significant difference (*P*< 0.05) when compared with the omeprazole (group 3) using one-way ANOVA followed by Tukey-Kramer Multiple Comparisons Test.

As shown in Table 1, pretreatment with 50 mg/kg of *astragaloside IV* exhibited the highest inhibition of lesion area formation (70.79%), followed by omeprazole (68.57%), *astragaloside IV* of 10mg/kg (41.26%) and 1mg/kg (18.03%). Pretreatment with 50 mg/kg of *astragaloside IV* (group 6) prior to WRS elicited the most effective gastroprotection (P<0.05). The total lesion areas (mm^2^) and percent of lesion inhibition are represented in Table 1 and Fig 1.

### Astragaloside IV increases gastric juice pH value and mucus production

Gastric acid from rats in the lesion control group demonstrated a significant decrease in gastric pH (2.05±0.09), creating a highly acidic and mucolytic environment. In contrast, pretreatment with indicated dosage of *astragaloside IV* or omeprazole was found to restore the pH value to the level observed in the control group (pH = 2.84±0.08). Although the efficacy of suppressing gastric acid secretion is not as effective as omeprazole, pretreatment with *astragaloside IV* induced a dose-dependent elevation gastric pH (Table 1 and Fig 1).

Gastric mucus acts as an important protective barrier against chemical or mechanical insults on the mucous membrane. Gastric mucus production in the experimental groups was estimated using a precision electronic balance. The measurements are presented in Table 1 and Fig 1. The apparent increase in gastric mucus production in *astragaloside IV* pretreated groups could offer protection against subsequent WRS-induced gastric injury. There was no statistical difference in gastric mucus production between group 6 (50 mg/kg of *astragaloside IV*) and the omeprazole treated group (P> 0.05).

### Examination of gastric pathology

**Fig 2:**
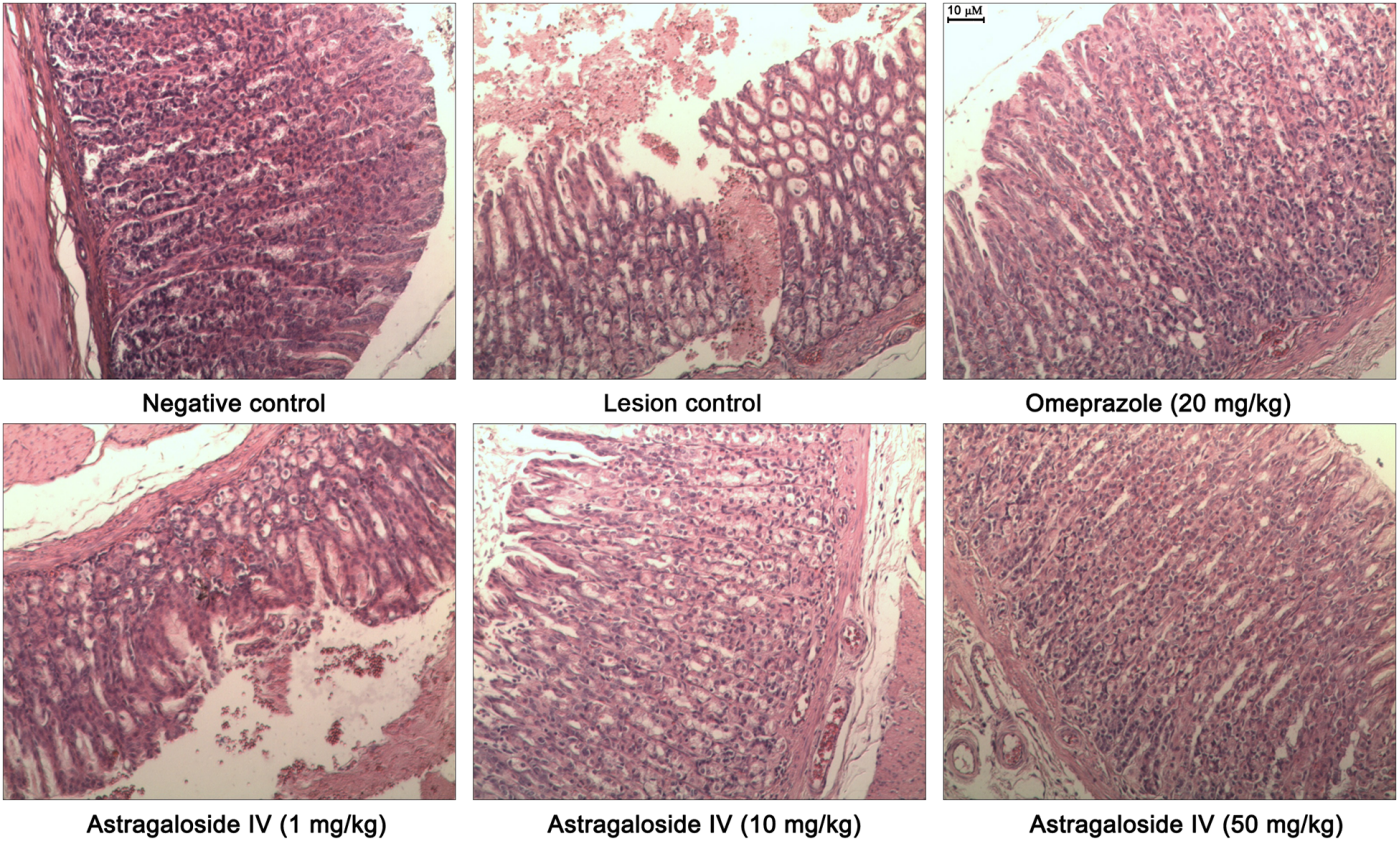
Representative photomicrographs of gastric mucosa in rats (H&E staining, ×100). In rats from negative control group, normal gastric tissue has intact gastric pits and glands. Rats exposed to WRS (lesion control) have severe mucosal erosion and submucosal edema with macrophage and eosinophil infiltrates. In rats pretreated with Omeprazole (20 mg/kg), inflammatory exudates and edema can be detected in submucosal. Rats pre-treated with *astragaloside IV* have a reduction in submucosal edema and leukocyte infiltration in a dose- dependent manner (1–50 mg/kg).

As shown in Fig 2, gastric samples from the negative control group exhibited histological features of normal gastric architecture with gastric pits and glands. In contrast, animals exposed to WRS exhibited mucosal erosion and submucosal edema with macrophage and eosinophil infiltrates that separates the mucosa and muscular is mucosal layer. Furthermore, the loss of normal glandular architecture of the mucosa was also observed (Fig 2, lesion control).

In the animals pretreated with omeprazole (20mg/kg), the gastric mucosa and gastric glands are neatly arranged. However, fewer inflammatory exudates and dilated capillaries can be detected.

Histomorphological examination indicated that the *astragaloside IV* elicited gastroprotective effects in a dose-dependent manner. Sections of the gastric mucosa of animals that received 10 mg/kg of *astragaloside IV* demonstrated a reduction in submucosal edema and leukocyte infiltration. The rats pretreated at 50mg/kg shown that the gastric mucosal structure is clear, the gland is neatly arranged, submucosal capillary mild expansion, the inflammatory cell is not obvious (Fig 2, lower right).

### Astragaloside IV induced increase in SOD along with decline in MDA, TNFα and MCP1 levels in gastric homogenate

**Fig 3:**
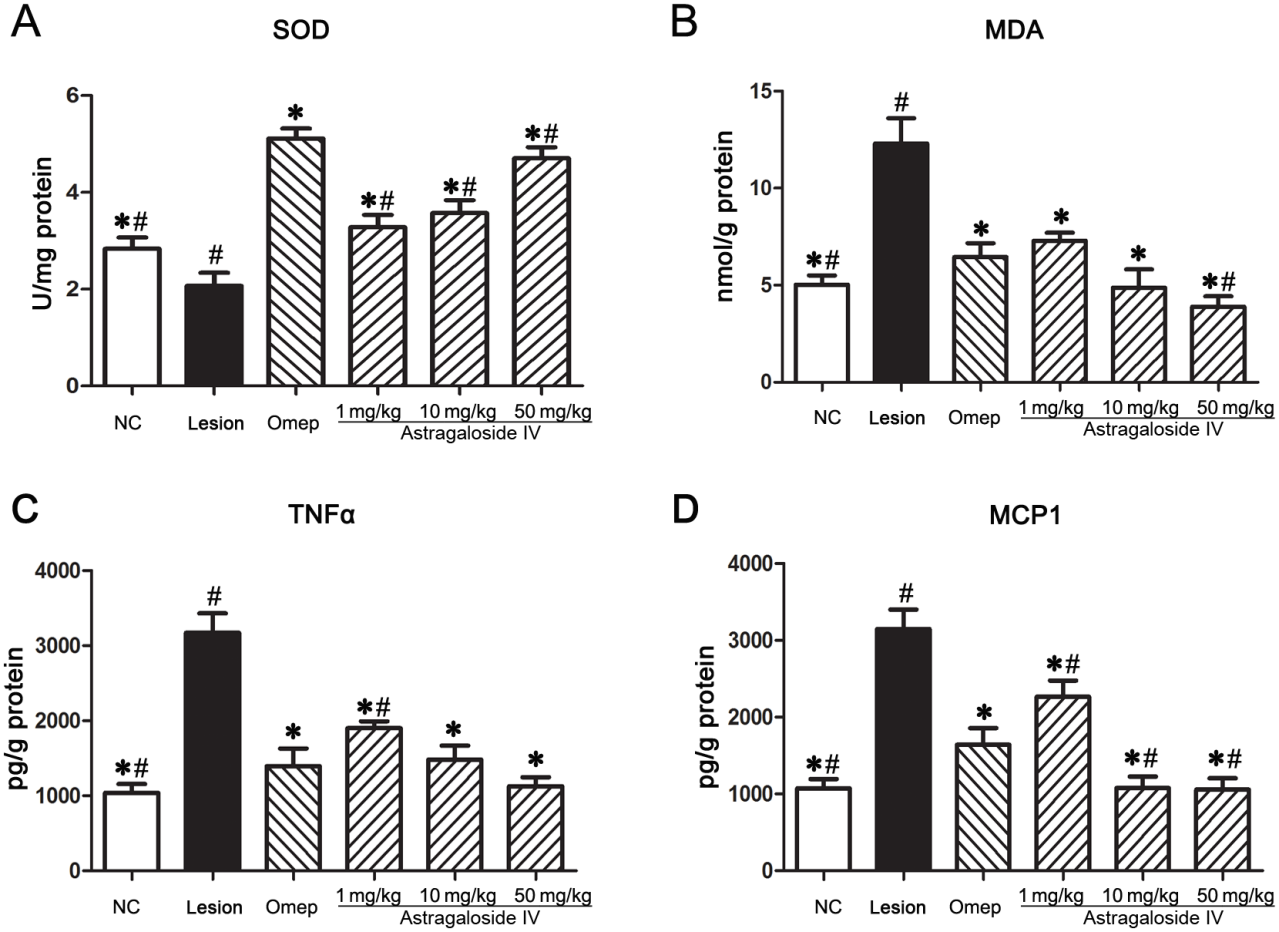
The effects of *astragaloside IV* on the SOD, MDA, TNFα and MCP1 response level in gastric homogenate. The SOD, MDA, TNFα and MCP1 response level were quantified using ELISA. All results expressed as mean ± standard error of mean. *Significant difference (*P*< 0.05) when compared with the lesion control (group 2). ^#^Significant difference (*P*< 0.05) when compared with the omeprazole (group 3).

Stress causes activation of the brain-gut axis, and as a result, promotes mucosal ischemia and mitochondrial leakage of superoxide anion (O^2-^), which further augments hydroxyl radical (OH.) production, with subsequent oxidative damage of macromolecules [20]. It has been shown that the antioxidative enzyme superoxide dismutatase (SOD) is correlated with elimination of reactive oxygen species (ROS) and free radicals [21]. ELISA analyses demonstrated that the SOD levels in the lesion control group were significantly reduced (P <0.05). However, animals pretreated with *astragaloside IV* show a marked increase in SOD in a concentration-dependent manner. At a concentration of 1 mg/kg, compared to saline, *astragaloside IV* treatment resulted in an evident augmentation in SOD levels. Upon increasing the concentration to 50 mg/kg, the SOD activity was significantly elevatedin comparison to thelesion control group (P <0.05, Fig 3A).

MDA is an end product of lipid peroxidation and acts as sensitive marker for evaluating free radical activity. We tested whether the observed elevation in SOD levels could be attributed to the MDA inactivation in response to *astragaloside IV* pre-treatment. Results of parallel experiments demonstrated that MDA levels were the highest in the lesion control group due to severe oxidative stress induced by WRS. In comparison to the lesion control group, treatment with 1 mg/kg of *astragaloside IV* resulted in a significant reduction of MDA levels from 12.30±1.31 to 7.29±0.41 nmol/g gastric homogenate. Increasing the concentration of *astragaloside IV* to 50 mg/kg resulted in a further reduction in MDA levels (P<0.01, Fig 3B). Together, this highlights *astragaloside IV*’s potential to inhibit lipid peroxidation and free radical production.

In addition to a decrease in the production of ROS, the infiltration of inflammatory cells is also believed to play a significant role during the pathogenesis of acute gastric lesions [22]. To gain insight into whether *astragaloside IV* could interrupt inflammatory signalling, we assessed the effect of *astragaloside IV* treatment on the production of proinflammatory cytokines. TNFα and MCP1 levels were measured by ELISA in the omeprazole and *astragaloside IV* treated groups compared to the lesioncontrol group (Fig 3C and 3D). TNFα and MCP1 expression levels significantly increased after 12 hours exposure to WRS in the lesion control group compared to vehicle controls (P<0.01). In contrast, TNFα and MCP1 expression exhibited a significant progressive decline when the experimental animals were pre-treated with increasing dose of *astragaloside IV* prior to WRS exposure. The reduction in TNF-α expression when treated with 50 mg/kg of *astragaloside IV* was comparable to the omeprazole pre-treatment group (P> 0.05, Fig 3C).

### Astragaloside IV induces HSP70 mRNA and protein expression following induction of acute gastric lesions

**Fig 4:**
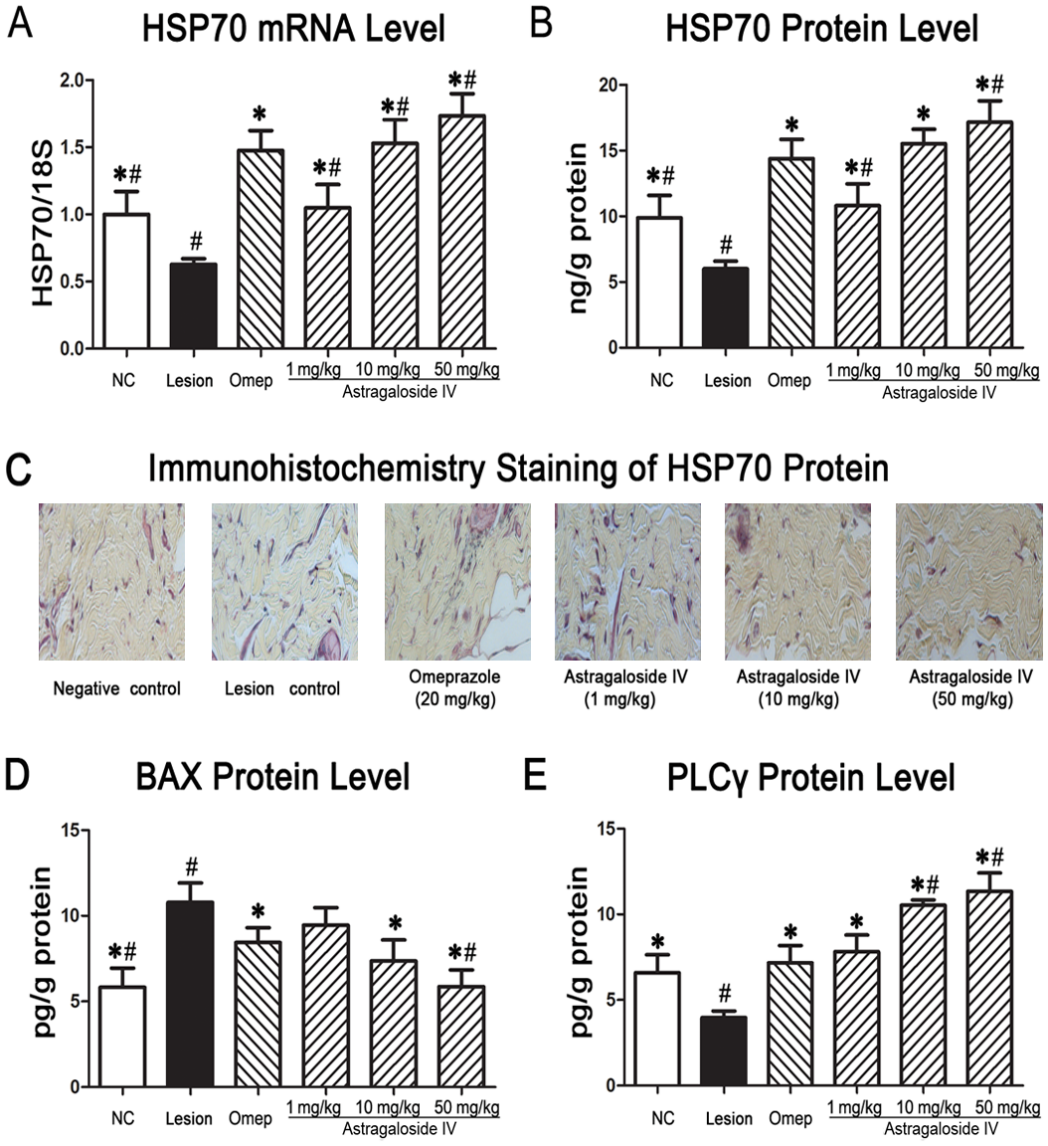
The effects of *astragaloside IV* treatment on the HSP70 gene expression and HSP70, BAX and PLCγ response levels. HSP70 gene expression levels were quantified using qRT-PCR. HSP70, BAX, and PLCγ response level were quantified using ELISA. Immunohistochemical analysis demonstrated the expression of HSP70 proteins (Magnification 20x). All results are presented as mean ± standard error of mean.*P< 0.05*vs*.the lesion control,^#^*P*< 0.05*vs*. the omeprazole group using one-way ANOVA followed by Tukey-Kramer Multiple Comparisons Test.

HSP70, a member of the heat hock protein family, has been shown to have a protective role against gastrointestinal diseases including gastric ulcers. In the intact gastric mucosa of vehicle rats, low HSP70 mRNA and protein expression was detectable, indicating a low basal HSP70 activity (Fig 4A and 4B). In the gastric lesion control group, HSP70 mRNA was significantly reduced compared to the vehicle control group. However, pre-treatment with *astragaloside IV* prior to WRS exposure induced a dose-dependent increase in HSP70 mRNA expression (Fig 4A). Pretreatment with 10 mg/kg and 50 mg/kg of *astragaloside IV* elicited an equal or greater response in HSP70 expression compared to pretreatment with omeprazole.

Subsequent analysis of total HSP70 protein revealed that *astragaloside IV* upregulated HSP70 protein synthesis in a proportional, dose-dependent manner (Fig 4B). *Astragaloside IV* treatment at a concentration of 1 mg/kg induced an observed increase in HSP70 protein, restoring that to baseline levels. Increasing the concentration to 10 or 50 mg/kg resulted a further increase in HSP70 protein levels (P <0.05).

Immunohistochemical analysis with anti-HSP70 antibody further confirmed that pretreatment of animals with *astragaloside IV* produced a significant increase in HSP70 protein levels that could otherwise be downregulated upon exposure to WRS (Fig 4C).

It has been previously reported that inflammation or oxidative stress-mediated damage to the gastric mucosa suppresses the expression of HSP70 proteins, simultaneously enhancing the expression of Bax protein, a pro-apoptosis regulator [23]. ELISA analyses of BAX protein showed that Bax was upregulated in the lesion control group in comparison to the vehicle control group (Fig 4D). Pretreatment with *astragaloside IV* (10 and 50mg/kg) prior to WRS-induced gastric lesion caused significant downregulation of Bax protein expression compared to the lesion control group.

### Astragaloside IV-induced gastroprotective effects along with the activation of PLCγ but not ERK1/2

Bax plays an important role in initiating the apoptosis pathway, whereas both ERK1/2 and PLCγ are key mediators in apoptotic death of cells [24]. To investigate the underlying mechanisms of the antiapoptotic effect of *astragaloside IV*-induced gastroprotective actions, we examined the expression levels of activated PLCγ and ERK1/2. Surprisingly, the level of activated ERK1/2 was not altered by *astragaloside IV*, suggesting that *astragaloside IV* exerted its gastroprotective effect through a different pathway (data not shown).

Furthermore, we found that the PLCγ proteins levels from gastric homogenate in the lesion control group showed a marked decrease in comparison to the vehicle control group (3.97±0.14 pg/g vs. 6.59±0.37 pg/g protein, P<0.05). Pretreatment of rats with *astragaloside IV* (1 mg/kg) increased PLCγ levels significantly to mean values comparable to the omeprazole treatment group (7.82±0.97vs.7.17 ± 1.01 pg/g protein) as shown in Fig 4E. Following pre-treatment with higher concentration of *astragaloside IV* (10 and 50mg/kg) and subsequent gastric lesions induction, a further increase in PLCγ expression (10.53±0.32and11.34±1.08, respectively) was observed, suggesting that PLCγ is involved in the gastroprotective effects mediated by *astragaloside IV* (Fig 4E).

## Discussion

Stress-induced gastric lesions may be responsible for the acute and life-threatening gastrointestinal bleedings seen in the intensive care units. Thus, development of gastric protective therapies remains a major task for critical care research. It has been previously reported that *astragaloside IV* inhibited the formation of gastric mucosal damage following ethanol-induced gastric lesions in animal models and suggested that this effect could be partially linked to the effective increase in nitric oxide [25]. The results of the present study indicate that pretreatment with *astragaloside IV* protected gastric mucosa lesions, which may be attributed to its antioxidant activities, increased gastric mucus secretion, the pH level of gastric juice, SOD activity, decreased levels of MDA, lesion area, inhibition in inflammatory infiltration of the submucosal layer, up-regulation of HSP70 protein along with the expression of PLCγ. Together, our study demonstrates *astragaloside IV* attenuates acute gastric lesions provoked by stress in a similar efficacious manner compared to omeprazole.

The gastric mucosal layer plays a crucial protective barrier role in the defence against the assault from irritants and other agents such as bacterial products capable of causing inflammation [26]. The enhancement of mucus production by *astragaloside IV* treatment suggests it may impart additional protective properties on the integrity of the mucosal barrier. Consistently, another study also suggested that an increase in mucosal secretion induced by *Zinc Schiff*-derivative suppressed acute hemorrhagic mucosal lesions in rats [27].

Previous observations have suggested that HSP70, an important mediator of intracellular signaling and transport, plays a critical gastroprotective role in pathological situations involving physical trauma, immunological stress as well as exposure to various infections [28]. As molecular chaperones, HSP70 participates in the proteasome-mediated degradation of intracellular by products of inflammatory signaling and free radical damage, namely, nuclear factor κB (NF-κB), IL1β, ROS and Bax [29]. Results from other animal studies also demonstrated that artificial induction of HSP70 expression confers gastric mucosal defense against stressors, whereas exposure to such stress reduces HSP70 production [30]. Our current study corroborates with the finding that HSP70 expression is inversely related to the severity of gastric mucosal lesions (evaluated by lesion areas and gastric mucosal pathological changes). Our results also showed that *astragaloside IV* is a remarkable natural inducer of HSP70 in response to subsequent exposure to stress, this is in accordance with the evidence of the significance of HSPs in ameliorating stress-induced gastric lesions.

Furthermore, evidence suggests that HSP70 is a negative regulator of Bax proteins to halt the process of apoptosis during gastric lesion formation [31]. Similar reports provided direct genetic evidence that demonstrated enhancement of HSP70 induced gastric protection against indomethacin-induced lesions by inhibiting the activation of Bax proteins [32]. Our data demonstrated that *astragaloside IV*-treated rats displayed a heightened HSP70 response that preceded the down-regulation of Bax levels. This finding suggests of other possible mechanisms that may explain the gastroprotective effects of *astragaloside IV*, including anti-apoptotic properties involving suppression of proapoptotic proteins.

Interestingly, our results revealed that pre-treatment with a gradient concentration of *astragaloside IV* following induction of acute gastric lesions had induced a notable over-expression of PLCγ in comparison to lesion control counterparts (Fig 4). This finding suggests that the observed anti-apoptositic effect of *astragaloside IV* may be associated with PLCγ, but not ERK1/2, which both were necessarily required for regulating the cell cycle [33]. Other studies have shown that PLCγ protects against oxidative damage modulating the dynamics of NF-κB/Iκ-Bα, suggesting that PLCγ might be another important regulator in mediating the gastroprotective effects of *astragaloside IV* [34]. However, the exact downstreamtarget of PLCγ in HSP70-mediated apoptosis suppression needs to be further defined infuture studies.

## Conclusions

This study demonstrates that pretreatment with *astragaloside IV* attenuated the acute gastric mucosal lesions induced by WRS and the mechanism underlying the gastroprotection may be attributable to upregulation of HSP70 along with its antioxidant, anti-inflammatory and anti-apoptotic action as well as the enhancement of the gastric mucosal barrier. This study has provided a novel way for treating stress-induced gastric lesions with *astragaloside IV*, a Chinese medicinal extraction. Future studies should be aimed at defining the bioactive components responsible for the gastroprotective mechanisms of *astragaloside IV* indicated in this study.

## Abbreviations

Bax: BCL2-associated X protein
BOW: body of weigh
ELISA: Enzyme-linked immunosorbent assay
ERK1/2: extracellular signal-regulated kinase 1/2
HSP70: heat-shock protein 70
MCP1: monocyte chemotactic protein 1
MDA: malondialdehyde
NFκB: nuclear factor κB
PBS: phosphate buffer saline
PLCγ: phospholipase Cγ
SOD: superoxide dismutase
TNFα: Tumour necrosis factors-α
WRS: water immersion and restraint stress
